# Impact of the Invasive Alien Macrophyte *Ludwigia hexapetala* on Freshwater Ecosystems: Evidence from Field Data

**DOI:** 10.3390/biology12060794

**Published:** 2023-05-31

**Authors:** Emanuele Pelella, Beatrice Questino, Beatrice Luzi, Flaminia Mariani, Simona Ceschin

**Affiliations:** 1 Department of Sciences, University of Roma Tre, Viale G. Marconi 446, 00146 Rome, Italy; bea.questino@stud.uniroma3.it (B.Q.); bea.luzi3@stud.uniroma3.it (B.L.); flaminia.mariani@uniroma3.it (F.M.); 2 NBFC—National Biodiversity Future Center, 90133 Palermo, Italy

**Keywords:** aquatic plant, non-native plant, freshwater habitat, biological invasion, ecosystem impact, plant diversity

## Abstract

**Simple Summary:**

Biological invasions, i.e., colonization and the spread of species outside their native range, are among the main threats to biodiversity conservation. The Central and South American macrophyte *Ludwigia hexapetala*, which is considered invasive in Italy, can grow both in slow-flowing waters and along the banks of lakes, rivers, and canals. In this study, field data were collected to assess the impact of this alien species on the environmental parameters and plant diversity of the invaded habitats. The results show that aquatic *L. hexapetala* populations, characterized by leaves floating on the water surface, alter the habitats they colonise by reducing the available light and the amount of dissolved oxygen in the water. Moreover, these populations negatively affect aquatic plants as increasing levels of invasion correspond to a decrease in plant diversity. Conversely, bank *L. hexapetala* populations do not have the same negative impact on bank plant diversity. In fact, some bank plants, such as the native *Phragmites australis* (common reed), may form a barrier to *L. hexapetala* invasion by producing extensive and dense populations. Based on this evidence, promoting the conservation of key aquatic and bank native plants that can counter *L. hexapetala* invasion could be a useful management practice in environments at risk of invasion by this alien species.

**Abstract:**

Biological invasions are a serious threat to biodiversity conservation, especially in freshwater ecosystems. The American macrophyte *Ludwigia hexapetala*, which colonizes both the aquatic and bank habitats of lakes, rivers, and canals, is invading many waterbodies in Europe, becoming an increasingly worrisome threat in several European countries, including Italy. However, only fragmentary information is available on the actual impact of its invasion in these habitats. This study aims to collect field data from various freshwater habitats in central and northern Italy, to assess the possible impact of *L. hexapetala* on the environmental parameters and plant biodiversity of the invaded habitats. The results show that in aquatic habitats, dense floating *L. hexapetala* populations reduce the light levels and oxygen available in the water, consequently limiting the growth of other aquatic plants. Indeed, *L. hexapetala* populations negatively affect aquatic plant diversity, as an increase in *L. hexapetala* cover corresponded to a decrease in Simpson’s diversity index. In contrast, in bank habitats, *L. hexapetala* has no significant impact on plant diversity. Evidence suggests that some native species, such as *Phragmites australis*, which generally form compact populations along the banks, effectively counteract the invasion of *L. hexapetala*. This information may prove valuable for the environmental managers of those freshwater habitats where *L. hexapetala* invasion needs to be addressed and controlled.

## 1. Introduction

Freshwater habitats are highly susceptible to biological invasions because of their intrinsic vulnerability [1,2], and because they are often disturbed by numerous anthropogenic pressures, such as hydro-morphological alterations, water over-exploitation and water pollution; in fact, such disturbances generate unstable environmental conditions in these habitats [3,4], usually promoting the colonization and spread of alien plant species [5,6]. In freshwater habitats, several alien aquatic plants were found to be highly invasive and impactful, severely altering water properties and ecosystemic balances, and out-competing the native plants [1,6,7]. For example, the aquatic ferns *Salvinia molesta* D.S. Mitchell [8] and *Azolla pinnata* R.Br. [9], the water hyacinth *Pontederia crassipes* Mart. [10,11], and the American duckweed *Lemna minuta* Kunth [12,13,14], often form dense and widespread populations free-floating on the water surface that act as physical barriers by interfering with light penetration and gas exchanges. This behavior drastically reduces the amount of light and dissolved oxygen in the water, severely limiting the survival of aquatic plant and animal species in the invaded habitat.

One of the alien aquatic plants that may pose a serious threat to the conservation of freshwater ecosystems in Europe is the Central and South American plant *Ludwigia hexapetala* (Hook. and Arn.) Zardini, H.Y. Gu, and P.H. Raven, which is included in the Onagraceae family. *Ludwigia hexapetala*, introduced for ornamental purposes for its showy and numerous yellow flowers, was first reported in Europe in France around 1830 [15], from where it then spread to other European countries, also reaching Italy, where it was first reported in 1934 [16].

Fragmentary information on the impact of *L. hexapetala* on freshwater ecosystems comes mostly from studies carried out in France, which is also the European country most invaded by this alien species to date [17]. The invasiveness of *L. hexapetala* is mainly due to its ability to produce dense populations in water, negatively affecting water quality and establishing anoxic conditions that are unfavorable for other aquatic organisms [18,19,20,21]. Moreover, it can release allelopathic substances into the surrounding environment that hinder the seed germination, growth, and reproduction of other plant species [22,23,24]. The invasiveness of *L. hexapetala* is also related to its high ecological and morphological plasticity, which allows it to colonize both aquatic and bank habitats. In fact, *L. hexapetala* shows two different morphotypes, which are characterized by heterophylly and diversified growth forms [23,25]. In the early stage of its life cycle, it has an aquatic morphotype characterized by shorter stems, with rosettes of round leaves floating on the water’s surface. At a later stage, when it has colonized the bank habitat, it switches to a terrestrial morphotype, with elongated vertical flowering stems and lanceolate leaves [21,26,27]. It also produces two types of roots: branched roots, which grow downwards, anchoring the plant to the sediment, and spongy white roots, which grow upwards, acting as specialized structures (pneumatophores) for the aeration of tissues submerged in water [24,25,27,28].

Thiébaut et al. [29], and, more recently, Pelella et al. [24], have investigated the impact of *L. hexapetala* on single aquatic native plants, such as *Mentha aquatica* L. and *Utricularia australis* R.Br., respectively. However, the evidence from these studies comes from laboratory experiments and, thus, suffers from some limitations, such as short time frames, small volumes, and less complex biotic and abiotic interactions than those occurring under natural conditions [3,30]. Therefore, while taking into account the information gathered from these laboratory experiments, it is important to carry out field investigations that can provide a better understanding of the actual effects of *L. hexapetala* invasion on aquatic ecosystems. Therefore, this study aims to collect field data in some freshwater habitats of central and northern Italy to assess the impact of *L. hexapetala* on both environmental parameters and the native plant communities occurring in the invaded habitats.

## 2. Materials and Methods

### 2.1. Sampling Sites

**Figure 1:**
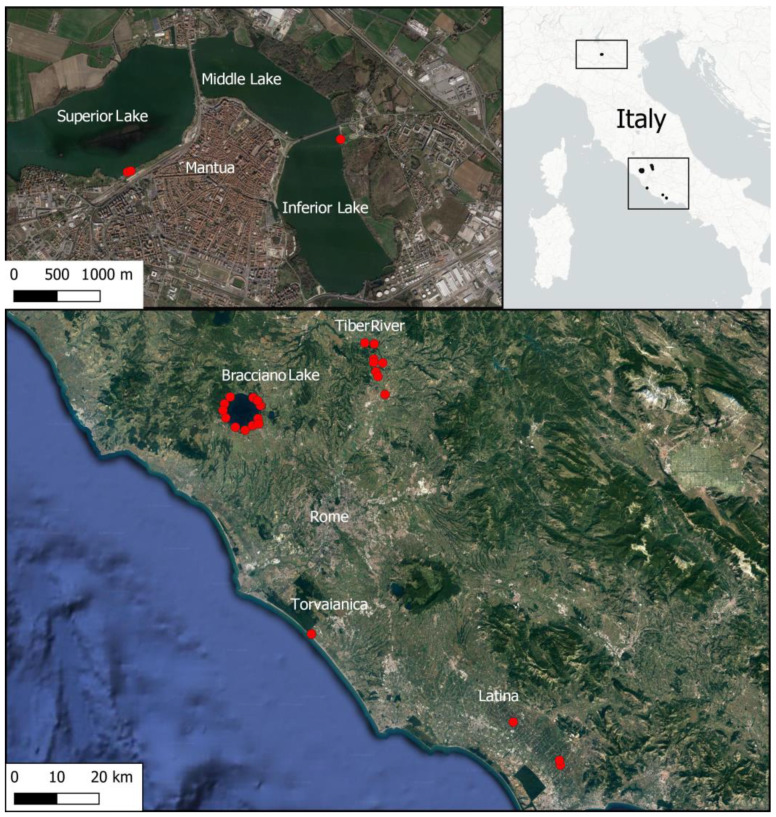
The study area. The dots represent the sampling sites.

A total of 50 relevés were carried out in different waterbodies (lakes, rivers, canals) in central and northern Italy, either where *L. hexapetala* has previously been reported [31,32] or that represent areas it could potentially colonize. Specifically, environmental and plant data were collected in the waters and along the banks of Bracciano Lake (22 relevés), Tiber River (12 relevés), Superior and Inferior Lake of Mantua (3 relevés), a canal in Torvaianica (8 relevés), and several canals in the province of Latina (5 relevés) (Figure 1).

Bracciano Lake is an oligo-mesotrophic, hard-water, volcanic lake in which *L. hexapetala* was originally reported in 2010, although it was initially confused with the congeneric *L. peploides* (Kunth) P.H.Raven [33]. After the drastic lowering of water levels in 2017 [34], the spread of this alien species along the banks increased, becoming a growing threat to local native biodiversity [35,36,37].

The Superior and Inferior Lakes are located along the Mincio River, adjacent to the city of Mantua and surrounded by urban areas. In these lakes, *L. hexapetala* has spread in the last decade [38].

The canal in Torvaianica is a drainage ditch surrounded by urban areas, where the invasion of *L. hexapetala* seems to be very recent, as it was first observed in the summer of 2021 [39].

In the province of Latina, there is an extensive network of canals carrying water that is used for irrigation and agriculture. Some of these canals have been invaded by *L. hexapetala*, including the Schiazza canal where the alien species was first reported in 2017 [31], representing the southernmost recorded population of *L. hexapetala* in Italy.

In the middle-lower course of the Tiber River, the presence of *L. hexapetala* has not yet been reported [31], although there are many aquatic and bank habitats along the river that could potentially be colonized due to having environmental characteristics compatible with the ecological requirements of the species [18,26]. Along this stretch of the Tiber River, several macrophyte communities that were similarly sampled by members of the same research group as this study [40], were considered reference communities that were *Ludwigia*-free.

### 2.2. Plant Sampling

Plant relevés were conducted between June and September 2022, corresponding to the main growing season of most macrophytes, including *L. hexapetala* [32,41].

**Figure 2:**
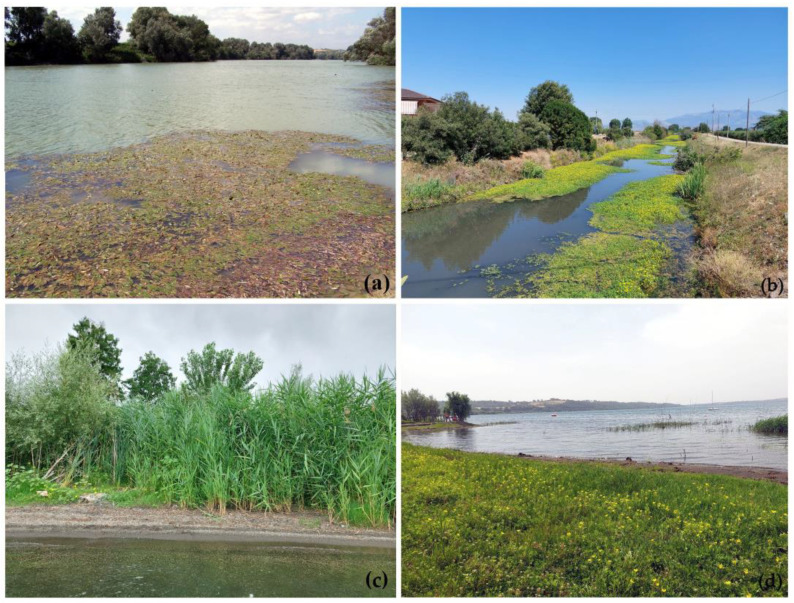
Sampling sites in aquatic (**a**,**b**) and bank (**c**,**d**) habitats without (**a**,**c**) or with (**b**,**d**) yellowish *L. hexapetala* flowering populations. Photographs show native aquatic populations of *P. nodosus* (**a**) and bank populations of *Phragmites australis* (**c**).

Samplings were carried out in both aquatic and bank habitats, at sites where *L. hexapetala* occurred with varying percentages of cover. To highlight the impact of *L. hexapetala* on invaded habitats, sites colonized by the alien species (29 sites) were sampled and compared with *Ludwigia*-free reference sites (21 sites) that were potentially compatible with the invasion of this species [18,26] (Figure 2). 

At each relevé, a standard 10 × 3 m rectangular area was delineated in the water or along the bank, with the longest side parallel to the bank. Aquatic and bank relevés were carried out separately to account for the different environmental characteristics and plant communities occurring in the two habitat types. For each relevé, the percentage of cover (%) and morphotype (aquatic/terrestrial) of *L. hexapetala*, as well as the presence and percentage of cover (%) of each occurring plant species were noted. The cover values were estimated by eye and were always estimated by the same researcher, to maintain data uniformity.

Simpson’s diversity index was calculated using the plant assemblage data, keeping the aquatic and bank plant communities separate. Then, the values of this index were correlated with *L. hexapetala* cover (%) to test whether an increase in the cover of the alien species (indicating an increasing level of invasion) would significantly affect local plant biodiversity. Simpson’s diversity index was calculated as follows:

1 − D = 1 − ∑*p_i_^2^*,

where *p_i_* is the proportional abundance of species *i*. 1 − D can range from 0 to 1, where 0 indicates no diversity and 1 indicates infinite diversity. Simpson’s diversity index was calculated using the R package, vegan [42].

### 2.3. Environmental Parameter Analysis

At each sampling site, samples were taken between 10 a.m. and 12 p.m., environmental parameters such as temperature, oxygen concentration, and light values were measured in the field three times. Air and water temperature (°C) and dissolved oxygen in water (mg/L) were measured with a multiparameter probe (Hach-Lange HQ40d). Light values in both air and water (μmol photon m^2^/s) were measured using a light meter (LI-250 light meter and LI 193 SA quantum sensor; LI-COR GmbH). At aquatic sites, to assess any reduction (%) in temperature and light between air and water, caused by the presence of *L. hexapetala* populations floating on the water surface, temperature and light were measured both in the air above *Ludwigia* populations and in the water below these floating plant mats. Similarly, at those bank sites where *L. hexapetala* occurred with its terrestrial morphotype, the temperature and light values were measured both above the *Ludwigia* populations and below at ground level. The percentage reduction (%) for these two parameters (P) was calculated as:

P(%) = (Po − Pu)/Po ∗ 100,

where Po represents the parameter value over the *Ludwigia* population, while Pu represents the parameter value under it.

### 2.4. Statistical Analyses

The calculated percentage differences in temperature and light between the measurements taken above and below the *L. hexapetala* populations were fitted against *L. hexapetala* cover, using linear mixed models (LMMs) to estimate the impact of the alien species on these parameters and employing different waterbodies as a random effect. Pseudo-R^2^ values were calculated and assumptions were checked against the model residuals. The same procedure was followed with the dissolved oxygen at aquatic sites. 

To assess the impact of *L. hexapetala* cover on plant diversity, linear mixed models (LMMs) were fitted using *L. hexapetala* percentage cover and Simpson’s diversity index as fixed effects, with waterbodies as random effects. The R packages nlme, lmer4, and lmerTest were used [43,44,45]. Pseudo-R^2^ (marginal and conditional) values were calculated for each model using the R package MuMIn [46]. Assumptions of normality and homoscedasticity were checked on model residuals. This analysis was carried out separately for aquatic and bank relevés since the two habitats have inherently different plant communities.

To further investigate the relationship between *L. hexapetala* and the other plants occurring in the invaded areas, an ordination analysis was performed. The aquatic community data was analyzed separately from the bank community data. The most common species at the sites, meaning those species found in at least 10% of the aquatic sites and 30% of the bank sites, were selected for the relevès. These percentages were chosen while taking into account the differences in the floristic patterns between the two types of environments. To determine whether a linear or unimodal ordination method was needed, detrended correspondence analysis (DCA) was performed on the log-transformed data, using the length of the first axis as the criterion. Since the DCA’s first axis was shorter than 4, a linear method was chosen. Specifically, the data were processed using principal component analysis (PCA), then the first three axes were selected. Ordination plots were made using ggfortify methods [47,48]. The ordination analysis included both those sites where *L. hexapetala* was absent and those sites with varying coverage of the alien species, to assess and understand any differences between the plant communities at non-invaded sites and at sites with different levels of *L. hexapetala* invasion.

All statistical analyses were performed using R software, version 4.2.1 [49].

## 3. Results and Discussion

### 3.1. Effect of L. hexapetala on Physical and Chemical Parameters

**Figure 3:**
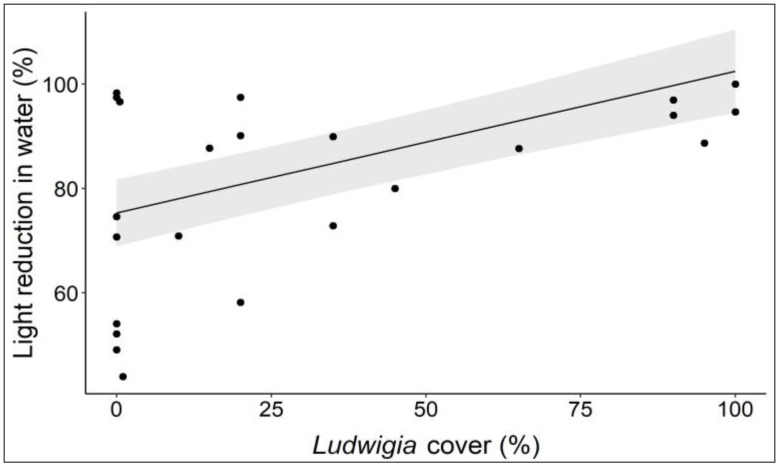
Light reduction (%) in aquatic sites with different levels of cover of *L. hexapetala*. Each dot represents a relevé; the black line represents the linear regression line; the grey bands represent 95% confidence intervals.

At aquatic sites, as the percentage of cover of *L. hexapetala* populations increased, there was a significant reduction (*p* < 0.001) in the light levels below the water surface (Figure 3). This means that *L. hexapetala*, by producing extensive populations floating on the water surface, reduces the availability of light in the water for other photosynthetic aquatic organisms. The difference in waterbodies explained 36% of the variance, which can be justified by considering that the waters belonging to the different waterbodies showed inherently different levels of turbidity. No significant correlation (*p* > 0.05) emerged between the percentage reduction in the temperature measured above and below the floating populations of *L. hexapetala* and the percentage cover of the alien plant, indicating that the floating mats produced by this species are not thick enough to prevent heat diffusion between the air and water.

**Figure 4:**
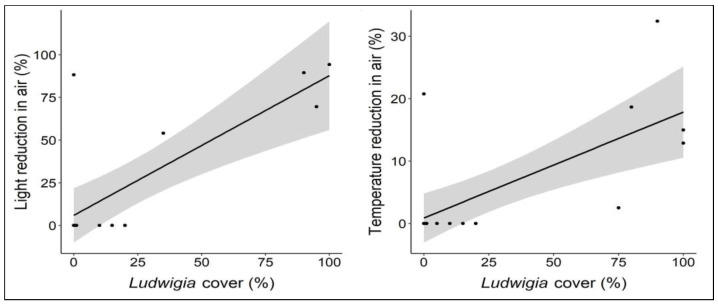
Light and temperature reduction (%) in bank sites with different levels of cover of *L. hexapetala*. Each dot represents a relevé; the black line represents the linear regression line; the grey bands represent 95% confidence intervals.

When analyzing the possible effect that bank populations of *L. hexapetala* may have on light and temperature, it was found that in both parameters, a significantly positive correlation (*p* < 0.001) was found between the percentage reduction in these two parameters and the percentage of cover of the *Ludwigia* populations (Figure 4). Indeed, due to its obvious morphological plasticity, *L. hexapetala* can produce large and dense populations not only in water but also along the banks of lakes, canals, and rivers. In this case, however, instead of growing horizontally, as is the case in the aquatic morphotype, *L. hexapetala* individuals grow primarily upward, producing bank populations that can reach 1 m in height [23,50]. Therefore, as their percentage of cover increases, these populations proportionally limit the passage of light and heat from the air to the soil surface. As a result, low light and heat conditions are established under these populations of *L. hexapetala*, limiting the growth of other plants.

**Figure 5:**
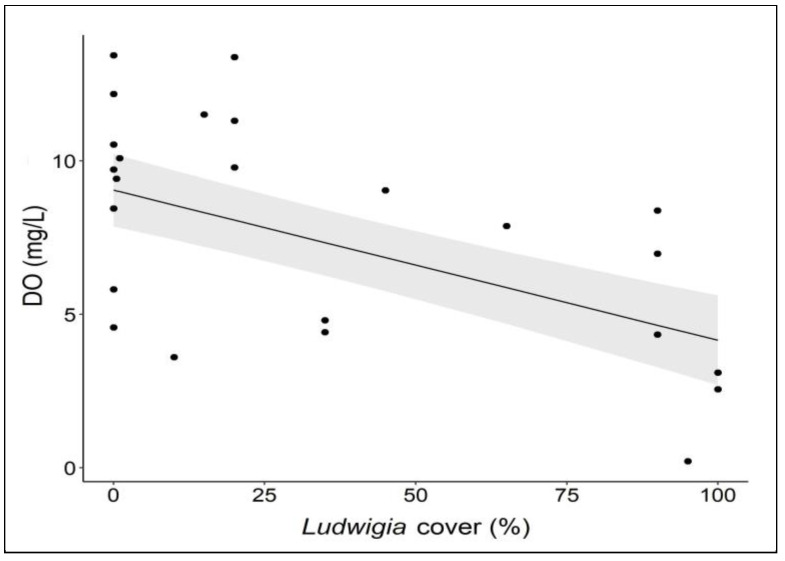
Dissolved oxygen (DO) in aquatic sites with different coverage levels of *L. hexapetala*. Each dot represents a relevé; the black line represents the linear regression line; the grey bands represent 95% confidence intervals.

Regarding the dissolved oxygen in the water, the different waterbodies explained 30% of the variance, as they showed inherently differently oxygenated waters. However, the dissolved oxygen decreased significantly (*p* < 0.01) with the increasing coverage of the aquatic populations of *L. hexapetala* (Figure 5). This result suggests that *L. hexapetala*, with its aquatic morphotype, adversely affects water oxygenation, due to the production of dense floating mats that interfere with the gas exchange at the water–air interface, leading to lower oxygenation in the water column beneath these populations [51]. In addition, it is able to produce pneumatophores that effectively capture most of the oxygen dissolved in the water [18,24,26,52], reducing the amount of oxygen available for other aquatic organisms.

### 3.2. Effects of Ludwigia hexapetala on Plant Communities

**Figure 6:**
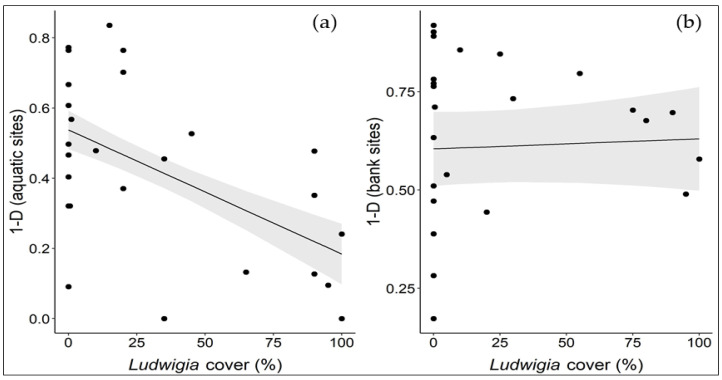
Influence of the different *L. hexapetala* coverage levels (%) on Simpson’s diversity index in aquatic (**a**) and bank (**b**) sites. Each dot represents a relevé; the black line represents the linear regression line; the grey bands represent 95% confidence intervals.

The results of the Simpson’s diversity index analyses show that *L. hexapetala* exerts different impacts on aquatic and bank communities. In aquatic sites, as the *L. hexapetala* coverage increased, there was a significant reduction in plant diversity (*p* < 0.01), regardless of the type of waterbody (Figure 6a). This means that *L. hexapetala* has a negative impact on the diversity of aquatic plant communities regardless of the type of freshwater habitat invaded (lake, river, canal), highlighting a high susceptibility of local aquatic communities to the invasion of this alien species. These results are in line with those of other studies on the impact of alien aquatic macrophytes on the abundance and diversity of native aquatic species [53,54].

In contrast, in the bank sites, *L. hexapetala* demonstrated no significant effect on bank plant diversity (*p* < 0.05) (Figure 6b). 45% of the variation among bank sites can be explained by the diversity of the waterbodies, which means that part of the differences in diversity among the plant communities sampled along the banks is linked to the inherent peculiarities of each waterbody, rather than to the presence of *Ludwigia* populations. In this case, it seems that the local bank communities are more resistant to *L. hexapetala* invasion, probably because these communities are generally more structurally compact than aquatic ones and are, thus, more likely to “build a wall” capable of counteracting the initial establishment and then spread of *Ludwigia*. Nobis et al. [55] also pointed out that invasive alien aquatic plants have a minimal influence on the species richness and plant cover of bank vegetation, compared to other more relevant drivers such as abiotic and anthropogenic factors. 

**Figure 7:**
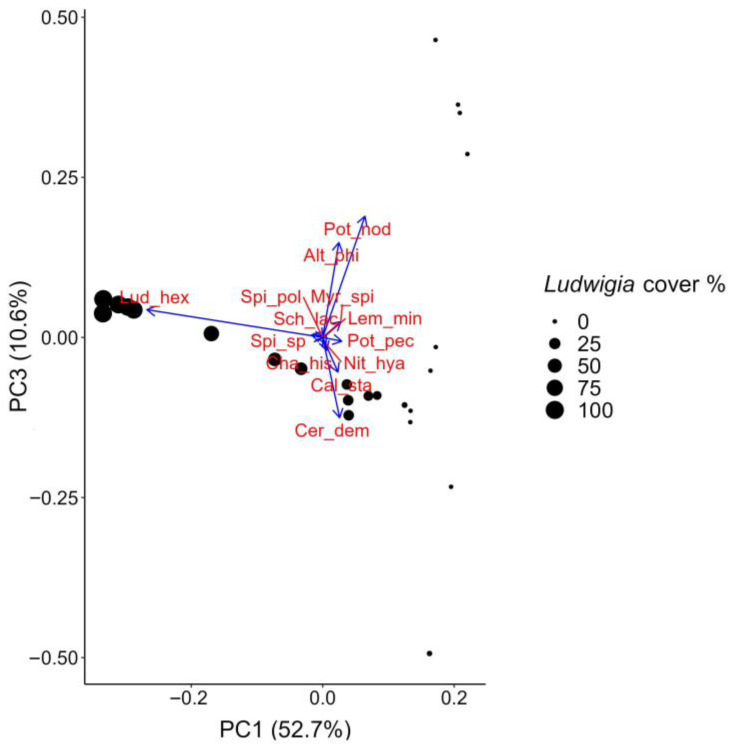
Principal component analysis on aquatic plant data. The ordination plot is based on the PC1 and PC3 axes. Blue arrows indicate species; black dots represent each relevé. The size of each dot represents the level of *L. hexapetala* coverage. Labels: Lud_hex = *Ludwigia hexapetala*; Pot_nod = *Potamogeton nodosus*; Pot_pec = *Potamogeton pectinatus*; Alt_phi = *Alternanthera philoxeroides*; Cer_dem = *Ceratophyllum demersum*; Cal_sta = *Callitriche stagnalis*; Cha_his = *Chara hispida*; Lem_min = *Lemna minor*; Myr_spi = *Myriophyllum spicatum*; Nit_hya = *Nitella hyalina*; Spi_pol = *Spirodela polyrhiza*; Spi_sp = *Spirogyra* sp.

As for the PCA of the plant data collected at aquatic sites, the PC1 and PC3 axes together explained more than 60% of the total variation (Figure 7). In the ordination space, *L. hexapetala* was completely isolated from other aquatic plants, showing an opposite distribution. In particular, in areas where *L. hexapetala* showed greater coverage, the other plants, especially some natives such as *Potamogeton nodosus* Poir., *Ceratophyllum demersum* L., *Callitriche stagnalis* Scop., *Chara hispida* L., and *Myriophyllum spicatum* L., were either absent or showed very low coverage, underlining how their presence seemed to be severely limited by the dominance of *L. hexapetala*. Indeed, this study shows that the presence of dense floating populations of *L. hexapetala* exerts an indirect impact on the growth of other aquatic plants by reducing the availability of light and heat and the levels of dissolved oxygen in the water. In addition to this type of impact, as shown in several studies, *Ludwigia* is capable of producing and releasing allelopathic substances into the water that strongly inhibit the seed germination and growth of other plants [22,23,24]. In contrast, the alien aquatic species *Alternanthera philoxeroides* (Mart.) Griseb. shows some degree of opposition to *L. hexapetala*; in fact, at those sites where *A. philoxeroides* was found, there was no *L. hexapetala*, although the latter was present at other (nearby) sites in the same body of water. This finding might suggest that some sort of exclusive interspecific competition between the two alien species was established.

**Figure 8:**
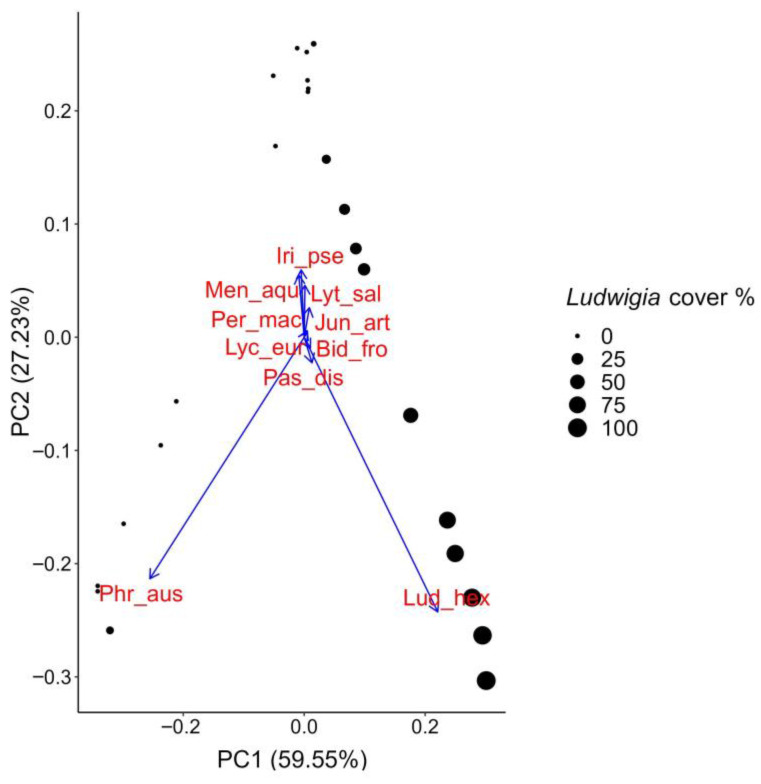
Principal component analysis on bank plant data. The ordination plot is based on the PC1 and PC2 axes. Blue arrows indicate species; black dots represent each relevé. The size of each dot represents *L. hexapetala* cover. Labels: Lud_hex = *Ludwigia hexapetala*; Bid_fro = *Bidens frondosa*; Iri_pse = *Iris pseudacorus*; Jun_art = *Juncus articulatus*; Lyc_eur = *Lycopus europaeus*; Lyt_sal = *Lythrum salicaria*; Men_aqu = *Mentha aquatica*; Pas_dis = *Paspalum distichum*; Per_mac = *Persicaria maculosa*; Phr_aus = *Phragmites australis*.

Regarding the PCA of the plant data collected at the bank sites, the PC1 and PC2 axes together explained more than 85% of the total variation (Figure 8). The most prominent native species in the ordination space is *Phragmites australis* (Cav.) Trin. ex Steud., which shows an opposite distribution along PC1 compared to *L. hexapetala*. In areas where *P. australis* dominates, the *L. hexapetala* populations are absent or have low coverage, and vice versa. This result might suggest the important counteracting role of this native species against the invasion of *L. hexapetala*. In fact, *P. australis* is a native plant that forms typically dense populations in the transition area between the aquatic and terrestrial habitats, which could potentially hinder the establishment along the banks of *L. hexapetala* propagules from nearby invaded aquatic areas. Other native bank species, such as *Mentha aquatica* L., *Lythrum salicaria* L., *Iris pseudacorus* L., and *Juncus articulatus* L., show some degree of opposition to *L. hexapetala*, increasing towards negative values of PC2, while the alien species increases towards positive values. However, the peculiar distribution of these species, which form a group that is isolated and separate from *L. hexapetala* in the ordination space, suggests that there is not a single species in opposition to it, as is the case with *P. australis*; rather, there appears to be a synergistic effect between different native bank species, which, when present with high levels of plant cover, are able to oppose the spread of this alien species.

Overall, these results could explain why *L. hexapetala* appears to be less able to colonize bank habitats than aquatic ones: along the banks, dense monospecific *P. australis* communities or aggregates of native species take on the role of opposing species, preventing or limiting the invasion of *L. hexapetala* in these areas. In fact, the impact of *L. hexapetala* on the diversity of bank plant communities has been shown to be not significant, unlike in the aquatic communities, which instead appear less resistant to invasion by this alien species.

## 4. Conclusions

Taking into consideration the fact that the evidence from this study is the result of a single survey campaign, the results obtained suggest that *L. hexapetala*-dominant populations seem to have an especially negative impact on aquatic habitats. Here, *L. hexapetala* seems to alter the waterbody’s physical and chemical properties, generating microenvironmental conditions that are hostile to the growth of other aquatic plants. The fact that *L. hexapetala* percentage cover is inversely correlated to the diversity of native aquatic plants further highlights the significant impact that this species has on these plant communities. In any case, the spread of *L. hexapetala* along bank habitats may encounter resistance; in fact, the native species *P. australis*, thanks to its dense populations that usually occur along the banks, seems to slow down or even prevent *L. hexapetala* colonization in these areas. Moreover, a more diversified native bank plant community could be more resistant to invasion by this alien species.

All this evidence can become particularly valuable for those environmental managers who, especially in the last decade in Italy, have been faced with the invasion of *L. hexapetala* unarmed, neither knowing the real competitive ability of this species against native plant communities, nor having full awareness of the effects that such a species can have on invaded freshwater ecosystems. Therefore, for better management of this invasive alien species, it will be necessary to carry out studies that can define its ecological requirements and identify any environmental factors that may limit its spread. Further investigations will also need to explore effective methods to contain or even prevent the invasion of *L. hexapetala*. In this case, mechanical control procedures combined with biological methods should be explored, also taking into consideration recent advances in biological control to combat the invasion of highly invasive aquatic species, such as *Lemna minuta* Kunth [13,56], *Pontederia crassipes* Mart., *Pistia stratioides* L., *Alternanthera philoxeroides*, and *Salvinia molesta* D.S.Mitch [57]. Although awareness of environmental problems resulting from biological pollution events should be the best defense against this threat [58], in an effort to prevent *L. hexapetala* invasion, management practices that also include conservation and implementation of native plants, such as *P. australis*, which have been shown to be more effective than others in efficiently countering the invasion of this alien species, should be promoted.

## Data Availability

The data presented in this study are available on request from the corresponding author.
